# Insight into the structure of black coatings of ancient Egyptian mummies by advanced electron magnetic resonance of vanadyl complexes

**DOI:** 10.5194/mr-3-111-2022

**Published:** 2022-07-13

**Authors:** Charles E. Dutoit, Laurent Binet, Hervé Vezin, Océane Anduze, Agnès Lattuati-Derieux, Didier Gourier

**Affiliations:** 1 Chimie-ParisTech, PSL University, CNRS, Institut de Recherche de Chimie-Paris (IRCP), 75005 Paris, France; 2 Université de Lille, CNRS, UMR8516-LASIRE, 59000 Lille, France; 3 Centre de Recherche et de Restauration des Musées de France (C2RMF), Palais du Louvre, 75001 Paris, France

## Abstract

Ancient Egyptian mummies from the Late Period to the Greco–Roman Period were covered by a black coating consisting of complex and heterogeneous mixtures of conifer resins, wax, fat and oil with variable amounts of bitumen. Natural bitumen always contains traces of vanadyl porphyrin complexes that we used here as internal probes to explore the nanoscale environment of V ${}^{4+}$ ions in these black coatings by electron nuclear double resonance (ENDOR) and hyperfine sub-level correlation spectroscopy (HYSCORE). Four types of vanadyl porphyrin complexes were identified from the analysis of ${}^{14}$ N hyperfine interactions. Three types (referred to as VO-P1, VO-P2 and VO-P3) are present in natural bitumen from the Dead Sea, among which VO-P1 and VO-P2 are also present in black coatings of mummies. The absence of VO-P3 in mummies, which is replaced by another complex, VO-P4, may be due to its transformation during preparation of the black matter for embalming. Analysis of ${}^{1}$ H hyperfine interaction shows that bitumen and other natural substances are intimately mixed in these black coatings, with aggregate sizes of bitumen increasing with the bitumen content but not exceeding a few nanometres.

## 1 Introduction

Mummies and wooden coffins, funerary artifacts and panel paintings in ancient Egypt were often covered with organic black materials, made of a heterogeneous mixture of natural substances such as fat, oil, wax, conifer or mastic tree resin, pitch, animal glue, plant gum and bitumen in variable proportions (Maurer et al., 2002; Buckley et al., 2004; Clark et al., 2016; Fulcher et al., 2021). All these components are characterized by a variety of molecular biomarkers identified mainly by gas chromatography–mass spectrometry (GC-MS) analysis. However, the presence or not of bitumen in these black materials has been the subject of controversy in the past due to the fact that the analytical protocols used were often not well adapted to the detection of bitumen, so that two opposite opinions have emerged among researchers analysing these black coatings: those who are doubtful about the presence of bitumen (Lucas and Harris, 1989; Buckley and Evershed, 2001; Davis, 2011) and those who claimed its presence (Spielmann, 1933; Rullkötter and Nissenbaum, 1988; Connan and Dessort, 1989; Colombini et al., 2000; Harrell and Lewan, 2002; Maurer et al., 2002). Thanks to the identification of specific biomarkers (hopanes, steranes) and radiocarbon analyses (bitumen has lost its ${}^{14}$ C), a consensus has emerged on the increasing presence of bitumen in embalming materials from the New Kingdom (ca. 1550–1070 BC) to the Ptolemaic/Roman period ending in the 4th century AD (Clark et al., 2016). Despite the inestimable contribution of GC-MS for revealing the molecular composition of these black coatings, this micro-destructive technique requires preliminary steps of fractionation and separation, which exclude any direct identification of bitumen, so that structural information on this black material cannot be obtained at the nanometre scale. This type of information requires the use of non-destructive techniques, which preserve the microstructure/nanostructure of the material, i.e. leave the samples intact. This is the case with magnetic resonance techniques because the low-frequency electromagnetic fields (radiofrequency for NMR and microwave frequency for electron paramagnetic resonance – EPR) penetrate the whole sample and deposit negligible energy in the material compared to the other spectroscopic techniques. Multinuclear magnetic resonance ( ${}^{1}$ H and ${}^{23}$ Na NMR) of mummified tissues is mainly used in the imaging mode (Mûnnemann et al., 2007; Özen et al., 2016) rather than the spectroscopic mode (Karlik et al., 2007), owing to the rather low sensitivity and spectral resolution of NMR for these highly disordered solid materials. EPR is the electronic equivalent of NMR and applies in the presence of unpaired electron spin density, i.e. with electron spin S≥1/2 . The spectroscopic resolution of EPR is optimal when the paramagnetic entities (transition metal ions, radicals) are magnetically diluted, which corresponds to defects and impurities in material (Bertrand, 2020). It is well known that oil and bitumen contain organic radicals and porphyrinic complexes of vanadyl (VO ${}^{2+}$ ) ions (V ${}^{4+}$ ion, 3d ${}^{1}$ configuration). These very stable paramagnetic molecules are present mainly in oil and bitumen and in asphaltene – the most refractory fraction of oil and bitumen – and can be considered molecular markers of bitumen which can be detected with high sensitivity by EPR spectroscopy (Rullkötter et al., 1985; Baker and Louda, 1986; Premovic et al., 1998; Ben Tayeb et al., 2015). Generally speaking, vanadyl porphyrin complexes (hereafter referred to as VO-P) are specific to oils and bitumen of marine origin (Barwise, 1990; Breit and Wanty, 1991; Lopez and Lo Monaco, 2017), while carbonaceous radicals (hereafter referred to as C ${}^{0})$ are present in all fossilized organic matter, whether of marine or terrestrial origin (Uebersfeld et al., 1954; Skrzypczak-Bonduelle et al., 2008; Bourbin et al., 2013), and even in the extraterrestrial carbonaceous matter (kerogen) of carbonaceous meteorites, the most primitive objects of the solar system (Binet et al., 2002). Recently, we showed that EPR analysis of VO-P complexes and C ${}^{0}$ radicals is a simple and non-destructive way (no sample preparation) of revealing the presence of bitumen in black coatings of Egyptian mummies, even in very small amounts (Dutoit et al., 2020). In addition to VO-P, these black coatings also contain non-porphyrinic VO ${}^{2+}$ complexes (hereafter referred to as VO-nP), with four oxygen ligands in a nearly square planar configuration (Dutoit et al., 2020). These VO-nP complexes are absent in the Dead Sea bitumen used by Egyptians and are found in black coatings containing natural substances in addition to bitumen. We hypothesized that this VO-nP could be localized at the interface between bitumen and other natural substances and result from the de-metalation of VO-P of the former followed by the complexation of VO ${}^{2+}$ by oxygenated functions of the latter (Dutoit et al., 2020).

**Figure 1:**
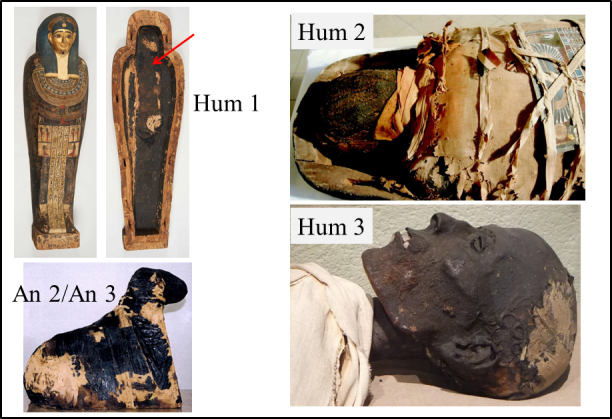
Origin of some samples of black matter studied by EPR: (Hum 1) coffin of Irethorerou (Ptolemaic dynasty), Art and History Museum, Narbonne, France (C2RMF76267, images cha17156 and cha17158). The black matter was sampled at the bottom of the coffin (arrow). ©C2RMF/Anne Chauvet. (Hum 2) Human mummy of the Late Period, Hieron Museum, Paray-le-Monial, France. The sample is a fragment of black matter covering the mummy. ©Hélène Guichard. (Hum 3) Head of the mummy from the Late Period, Chateau-musée, Boulogne, France. The sample was taken from the mummy's neck and reproduced with permission. ©Frédérique Vincent. (An 2/An 3) Ram mummy of the Late Period, Thomas Dobrée Museum, Nantes, France (Inv. D 961.2.140). The An2 and An3 samples are a fragment of black matter and a fragment of tissue with brown matter, respectively. ©Dépôt du musée du Louvre/Musée Dobrée-Grand Patrimoine de Loire-Atlantique.

**Table 1:** Origins of the samples of black matter.

Label	Origin
Hum 1	Anthropomorphic coffin, upper Egypt, Ptolemaic period (332–30 BC). Black coating in the bottom of the coffin.
Hum 2	Human mummy, Egypt, Late Period (end of the 4th century BC). Black coating covering the mummy.
Hum 3	Human mummy, Egypt, Late Period (21st–25th dynasties). Black matter taken from the neck of the mummy.
An 1	Ram mummy, upper Egypt, Late Period (672–322 BC). Black coating covering the mummy.
An 2	Ram mummy, upper Egypt, Late Period (664–332 BC). Black coating covering the mummy.
An 3	Ram mummy (the same as An 2). Tissue with brown matter covering the mummy.
An 4	Crocodile mummy, upper Egypt, Ptolemaic period. Black matter covering the skull.
Ref 1	Fragments of natural asphalt from the Dead Sea
Ref 2	Commercial powder of bitumen of Judea

However, the resolution of EPR spectra of VO-P in such highly disordered materials is limited by the fact that the weak hyperfine (hf) interactions with other nuclei, namely ${}^{1}$ H ( I = 1/2, 100 % abundance), ${}^{14}$ N ( I=1 , 99.6 % abundance) and ${}^{13}$ C ( I=1/2 , 1.1 % natural abundance), are unresolved. These unresolved hf interactions contain precious information about the structure of VO-P complexes, their environment and possibly the degree of alteration of the black matter. This information can be recovered by the indirect detection of NMR transitions of magnetic nuclei in the environment of the unpaired electron spin. By this means, VO ${}^{2+}$ of bitumen can be considered internal probes which “see” their nuclear spin environment in a non-destructive manner. Here we used electron nuclear double-resonance spectroscopy in continuous-wave mode (cw-ENDOR) for ${}^{1}$ H nuclei and hyperfine sub-level correlation (HYSCORE) spectroscopy for ${}^{14}$ N nuclei to study the same corpus of Egyptian black coatings as in the preliminary continuous-wave electron paramagnetic resonance (cw-EPR) study (Dutoit et al., 2020). We found that proton ENDOR is sensitive to the amount of bitumen in the black matter and to the size of bitumen aggregates, while HYSCORE of nitrogen reveals the presence of different types of VO-P complexes in the material. Special attention was given to the black coating of a human mummy of unknown origin (Hum 3) but whose EPR characteristics differ clearly from coatings of the other studied mummies (Dutoit et al., 2020).

## 2 Experimental procedures

### 2.1 Samples

The artifacts and mummies from which the samples of black coatings were collected, as well as their origins, are described in Fig. 1 and Table 1 and in more detail in Table S1 in the Supplement. The collected samples are shown in Fig. S1. Three fragments were taken from the coating of an anthropomorphic coffin (labelled Hum 1) dated from the Ptolemaic period (332–30 BC) and two human mummies: Hum 2 (end of the 4th century BC) and Hum 3 (presumably 25th Dynasty, 744–656 BC). Four fragments were taken from animal mummies dated from the same periods as the human mummies, among them three rams (An 1, An 2, An 3) dating from the Late Period (664–322 BC) and one crocodile (An 4) (Ptolemaic period, 332–30 BC). Their EPR spectra were compared with those of two pure bitumen samples: a fragment of natural asphalt from the Dead Sea (Ref 1) and a commercial powder of bitumen from Judea (Ref 2). Dead Sea asphalt was by far the most important source of bitumen supply in Egypt for the period corresponding to the samples studied in this work (Fulcher et al., 2021). The commercial Judean bitumen (Ref 2) having undergone preparatory treatments (undocumented), it allows us to test the stability of paramagnetic species and thus their relevance as a proxy for the study of black coatings. All samples (10–20 mg, Fig. S1) were inserted into quartz Suprasil EPR tubes. Each sample was weighed, and the EPR intensities were expressed per unit mass, $I_{s}/M_{s}$ , where $I_{s}$ is the measured amplitude of a perpendicular hf line for the VO-P spectrum and $M_{s}$ is the mass of the historical sample. Taking the Dead Sea asphalt, consisting of pure bitumen, as a reference, for which $I_{\mathrm{ref}}/M_{\mathrm{ref}}$ is measured, the normalized intensities of the VO-P spectra of historical samples are $100\times I_{s}M_{\mathrm{ref}}/(I_{\mathrm{ref}}M_{s})$ and given in wt % (see Fig. 4c). The absolute VO-P concentration corresponds to 10 ${}^{17}$ to 10 ${}^{18}$ V ${}^{4+}$ per gram of black matter, so the number of vanadyl complexes in EPR tubes is of the order of 10 ${}^{15}$ to 10 ${}^{16}$ .

### 2.2 EPR, ENDOR and HYSCORE experiments

cw-EPR measurements were performed at room temperature and at 100 K with a Bruker Elexsys E500 EPR/ENDOR spectrometer operating at about 9.6 GHz (X band) and 34 GHz (Q band), equipped with a high-sensitivity X-band 4122SHQE/0111 EPR cavity and a Q-band ER5106QTE resonator for both EPR and ENDOR. cw-ENDOR at Q band was used to measure the hyperfine interaction with ${}^{1}$ H nuclei of porphyrin ligands and their molecular environment (see Fig. 3a). The ENDOR spectra were recorded at 100 K by using a CF935 helium flow cryostat from Oxford Instruments. The radio waves were amplified by an ENI3100L amplifier, delivering a power of 10 W at the radio-frequency (rf) coil. The ENDOR signals were detected by a 25 kHz frequency modulation of the sweeping rf field, with a modulation depth of 100 kHz. The rf was swept in the range 45–60 MHz, centred at the proton Larmor frequency.

Pulse EPR experiments at X band were carried out with a Bruker ELEXSYS E580 spectrometer equipped with a Bruker cryostat “cryo-free” system. Two-pulse echo field sweep EPR spectra were recorded with the standard Hahn echo sequence π/2-τ-π-τ –echo. The resulting echo-detected absorption EPR spectrum (ED EPR) was pseudo-modulated to give a first-derivative ED EPR spectrum similar to the cw-EPR spectrum.

HYSCORE experiments were performed at 6 K with the pulse sequence π/2-τ-π/2-t1-π-t2-π/2-τ –echo, with pulse lengths of 22 and 44 ns for π/2 and π pulses, respectively. Due to the very small amount of VO-P in the EPR tubes (10 ${}^{15}$ to 10 ${}^{16}$ spins), it took at least 20 h to record each HYSCORE spectrum. For this reason a unique value of τ=200 ns was chosen as an optimum (Ben Tayeb et al., 2015). We checked by simulation that the value of τ had no impact on the separation of the ${}^{14}$ N dq–dq correlation peaks, which are important for the interpretation of the spectra (Fig. S9).

The spectra were recorded with 256×256 data points for the t1 and t2 time domains. The unmodulated part of the echo was removed by second-order polynomial subtraction. Final HYSCORE spectra were obtained by 2D Fourier transformation of the data set, using a Hamming apodization window function. EPR and HYSCORE spectra were simulated with the EasySpin toolbox for MATLAB (version 5.2.28) (Stoll and Schweiger, 2006).

## 3 Results and discussion

### 3.1 EPR spectra

**Figure 2:**
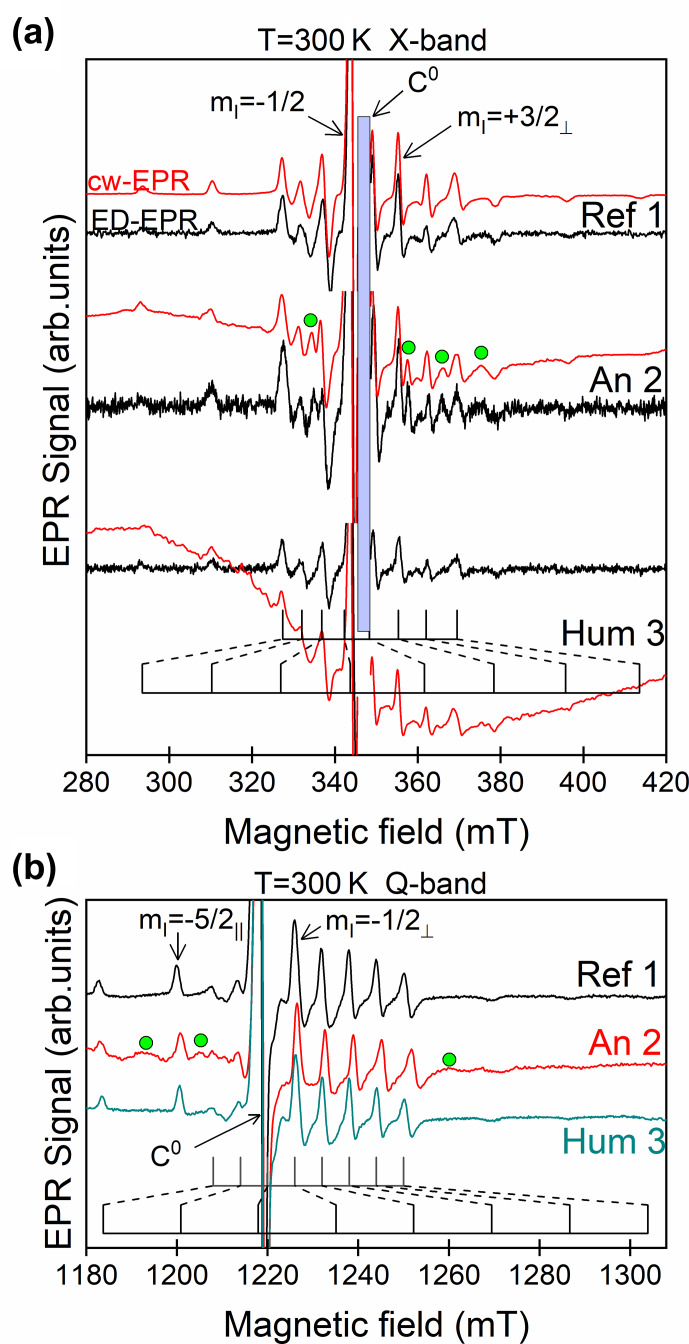
EPR spectra at room temperature of the black matter from samples Ref 1, An 2 and Hum 3. **(a)** cw-EPR spectra (in red) and pseudo-modulated ED EPR spectra (in black) at X band; the vertical blue area represents the position of the sharp C ${}^{0}$ signal that has been suppressed for the sake of clarity. **(b)** cw-EPR spectra at Q band. Some EPR lines of VO-nP complexes in An 2 are represented by green circles. The magnetic field settings for ENDOR (at Q band) and HYSCORE (at X band) experiments correspond to ${}^{51}$ V hf lines marked by arrows.

Examples of cw-EPR spectra at X band and Q band are shown in Fig. 2 for the Dead Sea asphalt (Ref 1) and the black coatings of mummies An 2 and Hum 3. The other spectra are given in Figs. S2–S4. All spectra show the two paramagnetic species classically present in bitumen and oil (Saraceno et al., 1961; Aizenshtat and Sundararaman, 1989; Premovic et al., 1998; Ben Tayeb et al., 2015) and recently identified by X-band EPR in black coatings of mummies (Dutoit et al., 2020): (i) organic radicals C ${}^{0}$ of asphaltene, represented by a single and intense line in the central part of the spectrum, with g=2.0037±0.0002 , and (ii) the spectrum of VO-P complexes embedded in the asphaltene, resulting from the hf interaction of the S=1/2 electron spin of V ${}^{4+}$ (configuration 3d ${}^{1})$ with the I=7/2 nuclear spin of the 100 % abundant ${}^{51}$ V isotope. This hf interaction gives two sets of 2I+1=8 lines characterized by g and hf parameters A given in Table 2. Parameters $g_{//}$ and $A_{//}$ correspond to VO-P complexes with the V–O bond oriented along the external field $B_{0}$ . The most intense central set of eight lines, with parameters $g_{⊥}$ and $A_{⊥}$ , corresponds to VO-P complexes oriented with $B_{0}$ lying in the porphyrin plane (and thus perpendicular to the V–O bond). The ${}^{51}$ V hf lines probed in HYSCORE (at X band) and ENDOR (at Q band) experiments are represented by arrows in Fig. 2a and b, respectively.

In addition, the black coatings of An 2 (Fig. 2), Hum 1, 2 and An 1, 3, 4 samples exhibit additional lines (some of them are marked by green circles in Figs. 2, S2 and S3). As the simulations show (Dutoit et al., 2020), other additional transitions are hidden under the VO-P transitions. All these lines belong to a non-porphyrinic vanadyl complex (referred to as VO-nP) characterized by $g_{//}=1.925\pm 0.003$ , $g_{⊥}=1.978\pm 0.003$ , $A_{//}=176(\pm 3)\times 10^{-4}$ cm ${}^{-1}$ and $A_{⊥}=70\pm (3)\times 10^{-4}$ cm ${}^{-1}$ , with oxygenated ligands in nearly square planar configuration (Dutoit et al., 2020). These complexes are thought to result from the interaction of bitumen with other bioorganic substances (resins, wax, fat) of the black matter. The only exception is the Hum 3 sample (the human mummy from the Boulogne museum, Fig. 1), which exhibits only the VO-P spectrum, like pure bitumen Ref 1 (Fig. 2) and Ref 2 (Fig. S2a). We concluded that mummy Hum 3 was covered with pure bitumen, which was confirmed by GC-MS analysis (Dutoit et al., 2020). In addition, the spectrum of Hum 3, An 1 (Fig. S2b, c) and, to a lesser extent, An 2 show a broad baseline distortion due to a ferromagnetic resonance (FMR) signal of iron oxide microparticles. This FMR signal, which does not give electron spin echo, can be eliminated by recording the echo-detected ED EPR, as clearly shown for An 2 and Hum 3 at X band (Fig. 2a).

**Table 2:** EPR parameters of VO-P complexes in the Ref 1, An 2 and Hum 3 samples.

Sample	g factors ( ±0.002 )	A (10 ${}^{-4}$ cm ${}^{-1})$
Ref 1	$g_{//}=1.959$	$A_{//}=157\pm 3$
	$g_{⊥}=1.980$	$A_{⊥}=54\pm 2$
An 2	$g_{//}=1.956$	$A_{//}=158\pm 3$
	$g_{⊥}=1.977$	$A_{⊥}=55\pm 2$
Hum 3	$g_{//}=1.957$	$A_{//}=155\pm 3$
	$g_{⊥}=1.978$	$A_{⊥}=52\pm 2$

The EPR parameters of VO-P complexes were deduced from the fitting of spectra at both the X and Q bands (Table 2). The slight differences of EPR parameters between the three samples fall within error bars of the simulations, except for parameter $A_{⊥}$ , for which the differences are clearly visible in the spectra at Q band (Fig. 2b).

The shape of EPR spectra of VO-P complexes (Fig. 2) is entirely controlled by the anisotropies of the g factor and of the strong hf interaction with the central ${}^{51}$ V nucleus, which reflect the electronic structure and the geometry of the complex. For this reason, the weak unresolved hf interactions with ${}^{1}$ H, ${}^{14}$ N and ${}^{13}$ C nuclei of the porphyrin ligand can only be revealed by hyperfine spectroscopy.

**Figure 3:**
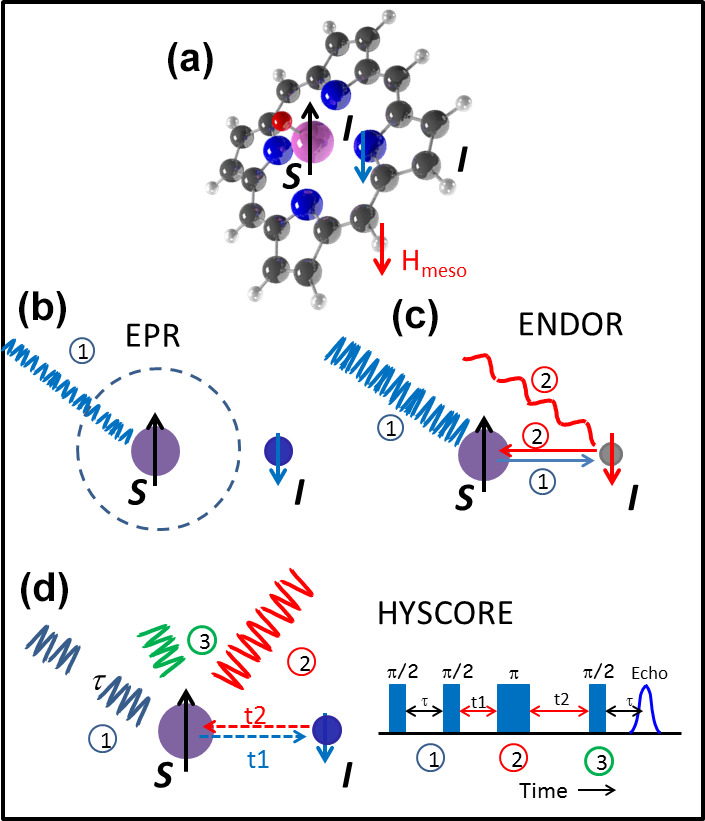
Principle of cw-ENDOR and HYSCORE spectroscopy. **(a)** Schematic structure of a porphyrin vanadyl complex, showing the electron spin S on vanadium and two nuclear spins I on nitrogen and on the bridging hydrogen H ${}_{\mathrm{meso}}$ . **(b)** EPR with a non-saturating microwave field, the dashed circle representing the limited resolution of EPR, which does not reach neighbouring ligand nuclei. **(c)** cw-ENDOR: a saturating microwave field modifies the populations of the nuclear spin state (step 1), while a strong rf field at nuclear frequency restores the nuclear populations, which in turn desaturates the EPR transition (step 2). **(d)** HYSCORE spectroscopy: a sequence of two π /2 microwave pulses separated by time τ induces a nuclear coherence in each electron spin state $m_{s}$ (step 1), and a π pulse after time t1 produces a transfer of nuclear coherence between the two electron spin states $m_{s}$ (step 2). After an evolution time t2, the nuclear coherences are transferred back to the electron coherence by a π/2 pulse, giving an electron spin echo at time τ (step 3).

### 3.2 ${}^{1}$ H ENDOR analysis

**Figure 4:**
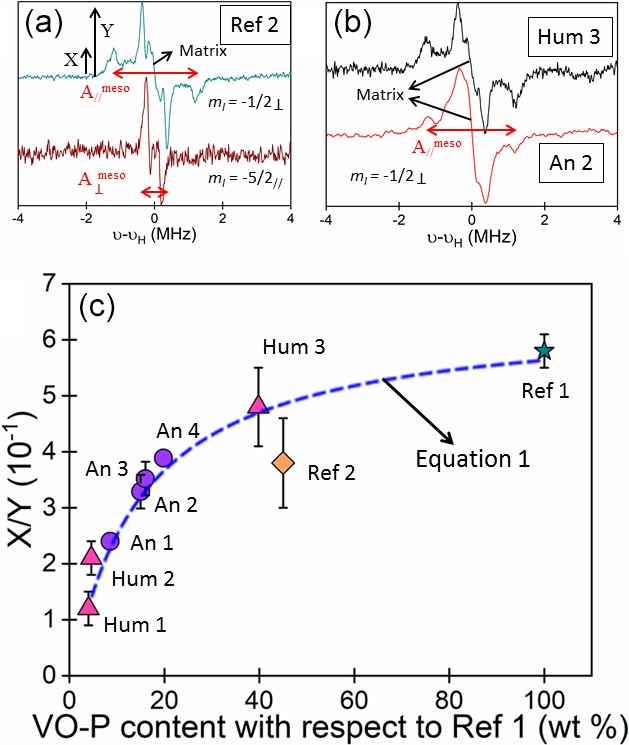
${}^{1}$ H ENDOR at Q band and at 100 K of VO-P complexes. **(a)** Bitumen of Judea (Ref 2), recorded by observing the $m_{I}=-1/2_{⊥}$ (1225.4 mT) and $-5/2_{//}$ hf lines (1197.9 mT) of ${}^{51}$ V (see Fig. 2b for the field settings). **(b)** Hum 3 and An 2 samples recorded at the $m_{I}=-1/2_{⊥}$ hf position (1230.6 and 1252.8 mT, respectively). **(c)** Variation of the ratio X/Y of ENDOR amplitudes versus the weight-normalized VO-P content of samples. The interrupted line was calculated from Eq. (1) with b=15 . The horizontal error bars correspond to the size of the data point.

EPR spectra are recorded with weak, non-saturating microwave radiation (Fig. 3a, b). In a cw-ENDOR experiment, an EPR transition is partially saturated at high microwave power and at a fixed magnetic field, which modify the population of the nuclear spin states. An rf field of frequency ν is then swept through the NMR frequencies of ${}^{1}$ H nuclei (Fig. 3c). The populations of nuclear spin states are modified at each nuclear resonance frequency, which are detected by a small increase in the EPR intensity (ENDOR enhancement). A typical ${}^{1}$ H ENDOR spectrum at Q band of the VO-P complex in pure bitumen (Ref 2) is shown in Fig. 4a, recorded at observing fields corresponding to the $m_{I}=-1/2_{⊥}$ and $-5/2_{//}$ ${}^{51}$ V hf lines (arrows in Fig. 3b). The ${}^{1}$ H signal is centred on the Larmor frequency $\nu_{H}$ of hydrogen (typically $\nu_{H}=51.9$ MHz for a magnetic field 1226 mT). As ENDOR spectra are recorded as a first derivative of the ENDOR enhancement with respect to the rf frequency, the spectral features occur at angular turning points characterized by ν – $\nu_{H}=\pm A_{//}/2$ and ν – $\nu_{H}=\pm A_{⊥}/2$ , where $A_{//}$ ( $A_{⊥})$ corresponds to the hf parameters of hydrogen atoms in VO-P complexes oriented such that the external field $B_{0}$ is parallel (perpendicular) to the V–H directions. The shape and parameters of the ENDOR spectrum depend on the nature of the C–H bond, on the V–H distance, and on the set of molecular orientations selected by the magnetic field settings. For an observing field set at the $-5/2_{//}$ EPR transition of VO-P, this corresponds to the selection of vanadyl complexes oriented with the V–O bond nearly parallel to the field vector $B_{0}$ , which is thus perpendicular to all the V–H ${}_{\mathrm{meso}}$ directions of the porphyrin plane (see Fig. 2a), so that only two ENDOR lines corresponding to $A_{⊥}$ are observed for these hydrogens (Fig. 4a, bottom). Alternatively, for a field setting at the $-1/2_{⊥}$ EPR transition of VO-P, the selected vanadyl complexes correspond to $B_{0}$ lying in the porphyrin plane and thus covering all possible angles with V–H ${}_{\mathrm{meso}}$ directions, so that both $A_{//}$ and $A_{⊥}$ ENDOR lines are observed in this case (Fig. 4a, top). The values $A_{//}=2.6$ MHz and $A_{⊥}=0.3$ MHz measured for VO-P correspond to the values known for the bridging C–H ${}_{\mathrm{meso}}$ bond of porphyrin in VO-P complexes (Biktagirov et al., 2017; Manniko and Stoll, 2019).

It must be noted that additional ENDOR features that would be expected at $A_{//}=1.3$ MHz and $A_{⊥}=0.4$ MHz for hydrogen of pyrrole groups (Gourier et al., 2010; Manniko and Stoll, 2019) are not observed in our ENDOR spectra. This indicates that all pyrrolic hydrogen atoms are substituted with alkyl groups, as known in the case of vanadyl geoporphyrins in oil and bitumen, such as vanadyl etioporphyrin (VO-EP) and vanadyl deoxophylloerythroetioporphyrin (VO-DPEP), for example (Fig. S6) (Dechaine and Gray, 2010). The narrow signal centred at $\nu_{H}$ , referred to as the “matrix” line (Kevan and Kispert, 1976), corresponds to more distant hydrogen atoms, which are weakly coupled to the electron spin by dipolar interactions. These include hydrogens of the alkyl side groups linked to pyrroles (mainly methyl and ethyl groups; see Fig. S6) and to hydrogens of the polyaromatic molecules of the surrounding asphaltene molecules but also to hydrogens of the bioorganic molecules (resins, waxes, and fats) of the black matter. In the case of pure bitumen (Ref 1 and Ref 2; see Figs. 4a and S5), only the alkyl side groups of porphyrin ligands and the asphaltene hydrogens contribute to the weak matrix ENDOR line.

Although EPR spectra of VO-P complexes are very similar in bitumen and all black coating samples (Figs. 2b and S4), their ENDOR spectra are somewhat different. The $A_{//}$ lines of H ${}_{\mathrm{meso}}$ atoms are well resolved in pure bitumen Ref 1 and Ref 2 and in the human mummy Hum 3 (Fig. 4b), while they broaden and decrease in intensity in black coatings of other samples (Figs. 4b and S5). Moreover, a pronounced matrix ENDOR signal centred at $\nu_{H}$ appears in all samples except pure bitumen (Ref 1 and *2*) and Hum 3 (Figs. 4b and S5).

This variation of the ${}^{1}$ H ENDOR spectrum can be quantified by the ratio X/Y , where X is the amplitude of the spectrum at the position of the $A_{//}$ line and Y is the amplitude at the position of the $A_{⊥}$ line, including the matrix line (see Fig. 4a). The variation of X/Y with VO-P content of the black coatings, normalized to the same mass of asphalt from the Dead Sea (Ref 1), is shown in Fig. 4c. We observed a regular decrease in X/Y with decreasing content of VO-P per unit mass of black matter, the VO-P content being related to the bitumen content. A value of X/Y≈5.6 is found for the pure natural bitumen (Ref 1), where the environment of VO-Ps consists mostly in asphaltene molecules. Mixing bitumen with increasing amounts of natural substances has three effects: (i) the decrease in VO-P content per unit mass of black matter (i.e. the decrease in EPR intensity of VO-P); (ii) an increase in the matrix ENDOR line (i.e. an increase in Y ) due to increasing amounts of hydrogen atoms of bioorganic molecules in the vicinity of VO-P; (iii) a broadening and weakening in the A ${}_{//}$ ENDOR lines of hydrogen H ${}_{\mathrm{meso}}$ (i.e. a decrease in their amplitude X ) resulting from a disorder (distribution of hf interaction values $A_{//})$ in the structure and environment of VO-P complexes. However, this disorder effect is not sufficient to modify the global square planar geometry of VO-P and has no visible effect on the shape and parameters of EPR spectra. The reference bitumen, Ref 2, deviates from the general trend in Fig. 4c. However, since this commercial bitumen has been processed, it may have undergone some undocumented chemical/physical treatment that could have modified its micro/nano structure. Nevertheless, the data for Ref 2 have been plotted in Fig. 4c only for the sake of comparison with historical and geological samples.

It is important to note that a matrix ENDOR line can occur only if the vanadium–hydrogen distances R are sufficiently small to give non-zero electron–proton dipolar interactions (i.e. R<5 –6 nm) (Kevan and Kispert, 1976; Stoll et al., 2005). We may conclude that only protons at distance R>0.6 –0.7 nm from the vanadium atom ( ∼ half the size of the porphyrin ligand) and R<5 –6 nm (limit for a non-zero effect on the matrix line) can contribute to the ${}^{1}$ H matrix line. This limited distance range for matrix protons has two consequences: (i) for a given proportion of bitumen and natural bioorganic substances, the amplitude Y of the matrix line should increase ( X/Y decrease) upon decreasing the radius $R_{A}$ of bitumen aggregates in the black matter (see Fig. S7 and Sect. S4 for details on the model) and reach a maximum amplitude for $R_{A}<5$ –6 nm (i.e. all VO-P complexes “see” hydrogens of natural bioorganic substances). (ii) For bitumen aggregates with a mean $R_{A}$ value, X/Y should decrease upon increasing content of natural substances, as more and more bioorganic hydrogen atoms are present in the vicinity of VO-P complexes. According to (i), X/Y should be nearly independent of VO-P content of the black matter if bitumen aggregates are large ( $R_{A}$ in the micrometer range or larger), because in this case, only the small fractions of VO-P close to the surface of bitumen aggregates “see” the bioorganic protons. The vast majority of VO-Ps being located in the volume of the bitumen aggregates, they should be insensitive to the presence or not of biomolecules around these aggregates because the hydrogens are at too great a distance to contribute to the matrix line. In this case the ratio X/Y should be nearly independent of the VO-P content of the samples and should be close to the value 0.6 for Ref 1, which is not observed experimentally. According to (i) and (ii), the fact that X/Y decreases regularly with decreasing VO-P content (Fig. 4c) for our body of black coatings (if we exclude the commercial bitumen Ref 2) indicates that the bitumen aggregates are very small ( $R_{A}<6$ –7 nm) in all cases and that X/Y depends only on the ratio bitumen / natural substances of the black matter.

A lower limit of the size $R_{A}$ of bitumen aggregates can be estimated from the fact that VO-P and radicals C ${}^{0}$ are spatially connected in asphaltene, with (VO-P)-C ${}^{0}$ distances not larger than 1–3 nm (Mamin et al., 2016). We previously showed that such spatial connection is conserved in bitumen of Egyptian black coatings (Dutoit et al., 2020). Consequently, we may roughly estimate that the sizes of bitumen aggregates in the studied black coatings lie in the range ∼1 nm < $R_{A}$ <∼6 –7 nm. Three scales of asphaltene aggregation were proposed in the Yen–Mullins model of asphaltene hierarchical structure (Mullins, 2010): the molecular ( ∼1.5 nm), the nanocluster ( ∼2.0 nm) and the cluster ( ∼5.0 nm) scales. It appears that the sizes of bitumen aggregates in black coatings correspond to the cluster scale of the Yen–Mullins model. Whatever the actual distribution size of asphaltene aggregates be, this ENDOR analysis shows that the bitumen and other natural substances are intimately mixed in black coatings. For such small sizes of bitumen aggregates, where the surface–volume ratio is high, this could also explain why a significant fraction of VO-P is transformed into oxygenated VO-nP complexes at interfaces between bitumen aggregates and natural substances (Dutoit et al., 2020).

The regular variation of the ENDOR shape factor X/Y with VO-P content for all historical samples (Fig. 4c) gives information about the nanostructure of these black coatings. This variation can be simulated with a simple model considering that X and Y amplitudes are both sums of contributions from protons of VO-P complexes and of the biomolecular layer around the bitumen aggregates, respectively, so that the ratio X/Y is given by (see Fig. S7 and Sect. S4 for demonstration)

1 $$\frac{X}{Y}=\frac{X_{\mathrm{VOP}}}{Y_{\mathrm{VOP}}}\times \frac{x+0.048\times b}{x+b},$$

where x is the vanadyl porphyrin content with respect to Ref 1 (the abscissa in Fig. 4c), and $X_{\mathrm{VOP}}$ and $Y_{\mathrm{VOP}}$ are the peak heights of the parallel and perpendicular components of the ${}^{1}$ H ENDOR spectrum of an isolated VO-P complex (measured in Ref 1 for $m_{I}=-1/2_{⊥}$ ). Parameter b in Eq. (1) is given by $b=aY_{m}/Y_{\mathrm{VOP}}$ , with $Y_{m}$ the peak-to-peak half-height of the matrix ENDOR line in each sample. The ratio $Y_{m}/Y_{\mathrm{VOP}}$ depends only on the ENDOR line shapes. Parameter a is a function of the bitumen content of the black matter and of the mean size of bitumen aggregates (see Sect. S4 for a demonstration). As $X_{\mathrm{VOP}}/Y_{\mathrm{VOP}}\approx 0.625$ for Ref 1, there is only one adjustable parameter b in Eq. (1). Experimental data were nicely fitted to Eq. (1) with b=15 (Fig. 4c). This good agreement shows that parameter b is a constant that is approximately independent of the samples (otherwise the experimental points in Fig. 4c would be scattered). As developed in Sect. S4, this constant value of b (except for the commercial bitumen Ref 2) means that, the larger the concentration of bitumen in the black matter, the larger the mean size of bitumen aggregates. This regular increase in size may be simply explained by the increasing coalescence of bitumen aggregates when they are less and less separated by the other bioorganic substances.

**Figure 5:**
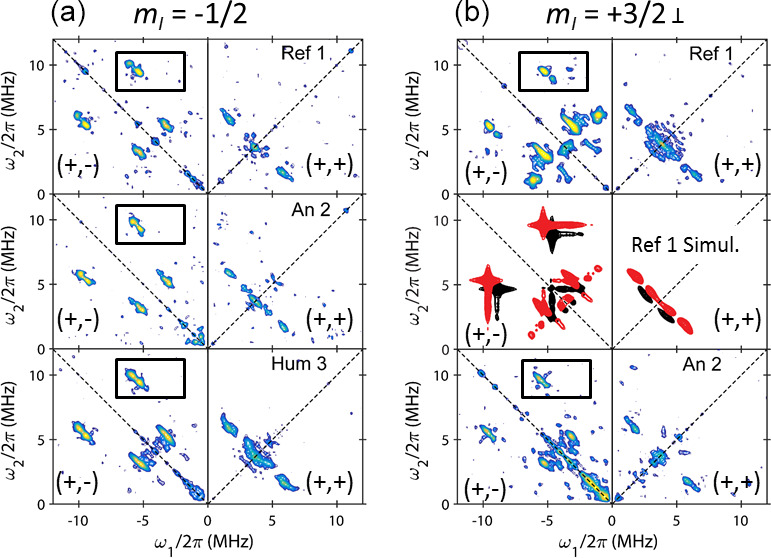
HYSCORE spectra at X band and at 6 K of VO-P in Dead Sea asphalt Ref 1 and in black coating of the An 2 and Hum 3 mummies, recorded by observing **(a)** the $m_{I}=-1/2$ (341,6 mT) and **(b)** the $m_{I}=+3/2_{⊥}$ ( ∼355,6 mT) hf lines marked by arrows in Fig. 2a. The correlation peaks described in more detail in Fig. 6 are delimited in rectangular boxes. Simulated spectra of VO-P1 (in red) and VO-P2 (in black) in the middle of **(b)**.

### 3.3 ${}^{14}$ N HYSCORE analysis

The very small variations of EPR parameters g and A of VO-P from one sample to another (Table 2) may suggest that several types of slightly different VO-P complexes are present in variable proportions in the bitumen component of black coatings. We used HYSCORE spectroscopy at X band to discriminate different types of VO-P by their ${}^{14}$ N hf interaction, with the perspective to use in the future these metallic complexes for getting information on the geographical origin of the bitumen and on its chemical and thermal treatment during the preparation of mummies. In pulse EPR spectroscopy (Schweiger and Jeschke, 2001), a spin echo is generated by a series of π/2 and π microwave pulses separated by controlled time delays (Fig. 3d). By varying these time delays, the echo intensity is modulated at frequencies of the hf interactions. HYSCORE spectroscopy is based on the pulse sequence π/2-τ-π/2-t1-π-t2-π/2-τ –echo, where τ is the delay between the first and second π/2 pulses. The first π/2 pulse generates an electronic coherence (a mixing of the two $m_{s}=\pm 1/2$ states) and the second π/2 pulse after time τ transfers the electronic coherence to nuclear coherences (mixing of $m_{I}$ states). After an evolution time t1 , a π pulse transfers the nuclear coherence from one $m_{s}$ state to the nuclear coherences of the other $m_{s}$ state, which creates correlations between nuclear transitions of these two $m_{s}$ states. After another evolution time t2 , a third π/2 pulse transfers the nuclear coherence back to the electronic coherence for detection, which generates an electron spin echo after time τ . The echo intensity is measured for the two times t1 and t2 , which are varied stepwise at constant τ value. The 2D frequency plot (HYSCORE) is obtained by 2D Fourier transformation of the data set in the time domain.

**Figure 6:**
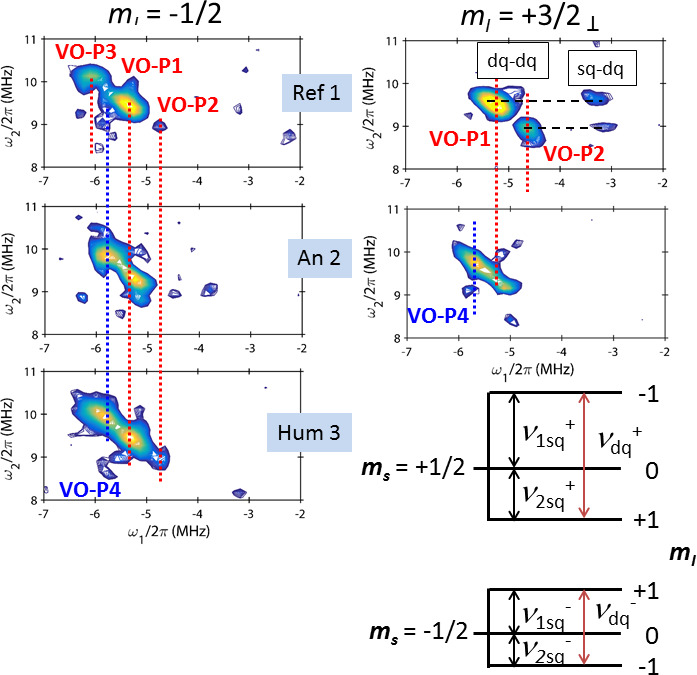
Portions of the HYSCORE spectra of Ref 1, An 2 and Hum 3 corresponding to dq–dq and dq–sq correlation peaks, where dq and sq represent double-quantum and single-quantum transitions, respectively. The diagram represents the spin states and the different nuclear transitions for a S=1/2 , I=1 spin system.

The correlations between nuclear transitions in the two $m_{s}=\pm 1/2$ states appear as cross peaks in the 2D frequency plot which are distributed in two different quadrants ( + , +) and ( + , - ) corresponding to $\omega_{2}>0$ , $\omega_{1}>0$ and $\omega_{2}>0$ , $\omega_{1}<0$ , respectively (Schweiger and Jeschke, 2001). For an electron spin S=1/2 interacting with an I=1/2 nuclear spin such as ${}^{13}$ C and ${}^{1}$ H, we expect two cross peaks in each quadrant, which take the shape of ridges perpendicular to the diagonal $\omega_{1}=\omega_{2}$ for anisotropic hf interactions in disordered materials (Schweiger and Jeschke, 2001). The spectrum is more complicated for a nuclear spin I=1 such as ${}^{14}$ N, which can give up to 18 cross peaks and ridges in each quadrant (Dikanov et al., 1996). The situation is even more puzzling if the electron spin is coupled with several nuclear spins (which indeed is the case with VO-P) because such multi-spin systems can give additional zero- and multi-quantum coherences as well as suppression effects (Stoll et al., 2005). Fortunately, many of these spectral features are too weak to appear in the 2D plot, so that the HYSCORE spectra remain interpretable (Reijerse et al., 1998; Garcia-Rubio et al., 2003; Dikanov et al., 2004). Representative HYSCORE spectra for samples Ref 1, An 2 and Hum 3 in the frequency range 0 to ±12 MHz are shown in Fig. 5. The HYSCORE spectrum of Ref 2 is reported in Fig. S8. We could not obtain acceptable HYSCORE spectra of Hum 1, Hum 2 and An 1 because of their low amount of VO-P. The spectra were recorded from two field settings in the EPR spectra (arrows in Fig. 2a): the $m_{I}=+3/2_{⊥}$ hf line (Fig. 5b), which selects VO-P complexes oriented with their V–O bond perpendicular to the external field $B_{0}$ (i.e. $B_{0}$ lies in the porphyrin plane), and the strong $m_{I}=-1/2$ hf line (Fig. 5a), whose position is almost independent of the field orientation (no selection of molecular orientations). Since our goal was to explore the diversity of VO-P complexes, we focused the spectral analysis on the narrow and relatively intense correlation peaks highlighted by the black boxes in the ( + , - ) quadrant of Fig. 5, which are represented in more detail in Fig. 6 for Ref 1, Hum 3 and An 2 and in Fig. S8 for Ref 2.

The energy-level diagram in Fig. 6 describes the spin states and the corresponding nuclear transitions for an S=1/2 , I=1 system. The frequencies for the single-quantum ( $\Delta m_{I}=1$ ) and double-quantum ( $\Delta m_{I}=2$ ) transitions, referred to as sq and dq transitions, respectively, are given by (Reijerse et al., 1998; Dikanov et al., 2004)

2 $$\begin{array}{l} \nu_{1\mathrm{sq}}^{\pm }=\frac{A}{2}\pm \nu_{N}+\frac{3Q}{2}+(\mathrm{second}-\mathrm{order}\;\mathrm{terms}),\\ \nu_{2\mathrm{sq}}^{\pm }=\frac{A}{2}\pm \nu_{N}-\frac{3Q}{2}+(\mathrm{second}-\mathrm{order}\;\mathrm{terms}), \end{array}$$

3 $$\nu_{\mathrm{dq}}^{\pm }=A\pm 2\nu_{N}+\frac{A^{\left(2\right)}}{\left(A/2\right)\pm \nu_{N}},$$

where $\nu_{N}$ is the nuclear Zeeman frequency and A and Q are the hf interaction and the quadrupolar interaction, respectively, of ${}^{14}$ N nuclei for a given field orientation. The full expressions including second-order terms in $\nu_{\mathrm{sq}}$ and their estimation are given in Sect. S7. The widths and positions of dq transitions (Eq. 3) are determined by the weak anisotropy of the ${}^{14}$ N–hf interaction A and by the second-order correction, while the widths and positions of sq transitions (Eq. 2) are mostly controlled by the strong anisotropy of Q (which is a traceless tensor) in addition to A and second-order corrections. For this reason, the sharp dq–dq correlation peaks, which depend only on A anisotropy to first order, have sufficiently high resolution and sensitivity to be used for the identification of various VO-P complexes. The quadrupolar interaction Q for each type of VO-P can only be determined by using sq frequencies (Eq. 2).

**Table 3:** Experimental (Exp) and simulated (Sim) hyperfine and quadrupolar parameters for ${}^{14}$ N nuclei (nm: not measured) in VO-P complexes.

Complex		$a_{\mathrm{iso}}$ (MHz)	T (MHz)	$Q_{zz}$ (MHz)	Samples	
VO-P1	Exp	-7.3	<0.1	0.94	Ref 1; Ref 2;	
					Hum 3; An 2	
	Sim	-7.3	0.1	1.0		
VO-P2	Exp	-6.5	0.2	1.1	Ref 1; Ref 2;	
					Hum 3	
	Sim	-6.6	0.2	1.0		
VO-P3	Exp	( ≈-7.3 )	nm	nm	Ref 1; Ref 2	
VO-P4	Exp	-6.8	0.6	nm	Hum 3; An 2	

Let us take the example of the HYSCORE of Dead Sea asphalt Ref 1, observed at the $m_{I}=+3/2_{⊥}$ field position and shown in the top right of Fig. 6. Two distinct dq–dq peaks are clearly observed, representing two different types of VO-P complexes, hereafter referred to as VO-P1 and VO-P2. The frequency coordinates of these dq–dq peak are [ -5.3 ; +9.5 ] MHz for VO-P1 and [ -4.6 ; +8.9 ] MHz for VO-P2, with an error bar of about ±0.1 MHz. The hf interaction A is directly obtained from Eq. (3):

4 $$A=\frac{2\nu_{N}\left(\nu_{\mathrm{dq}}^{+}+\nu_{\mathrm{dq}}^{-}\right)}{8\nu_{N}-\left(\nu_{\mathrm{dq}}^{+}-\nu_{\mathrm{dq}}^{-}\right)},$$

where the second-order term of Eq. (3) is naturally eliminated. With $\nu_{N}=1.1$ MHz at 355.7 mT, we obtained A=-7.28 MHz for VO-P1 and A=-6.6 MHz for VO-P2. The hf coupling was chosen negative on the basis of theoretical calculation on the VO-P complex in crude oil (Gracheva et al., 2016).

The vanadyl content of Ref 1 is sufficiently high to reveal two weaker sq–dq correlation peaks at [ -3.3 ; + 9.5] MHz for VO-P1 and [ -3.1 ; + 8.9] MHz for VO-P2 at the $m_{I}=+3/2_{⊥}$ field setting (Fig. 6). As an sq–dq peak correlates a dq transition $\nu_{\mathrm{dq}}$ of one $m_{s}$ state with an sq transition $\nu_{\mathrm{sq}}$ of the other $m_{s}$ state, all sq and dq frequencies of the spin diagram in Fig. 6 can be simply deduced by considering Eq. (2), which give $\nu_{1\mathrm{sq}}^{+}-\nu_{1\mathrm{sq}}^{-}\approx \nu_{2\mathrm{sq}}^{+}-\nu_{2\mathrm{sq}}^{-}\approx 2\nu_{N}$ to first order and $\nu_{1\mathrm{sq}}^{\pm }+\nu_{2\mathrm{sq}}^{\pm }=\nu_{\mathrm{dq}}^{\pm }$ . The results for VO-P1 and VO-P2 are shown in Fig. S11. Neglecting again second-order terms, the quadrupolar parameter Q can be estimated from Eq. (2) by $Q\approx \left(\nu_{1\mathrm{sq}}^{-}-\nu_{2\mathrm{sq}}^{-}\right)/3\approx \left(\nu_{1\mathrm{sq}}^{+}-\nu_{2\mathrm{sq}}^{+}\right)/3$ . The values for VO-P1 and VO-P2 are $\left|Q\right|\approx 0.47\pm 0.03$ MHz and $\left|Q\right|\approx 0.55\pm 0.02$ MHz, respectively (see Sect. S7 for the estimation of the second-order terms).

The ${}^{14}$ N–hf interaction is anisotropic, with two components $A_{//}$ and $A_{⊥}$ , corresponding to $B_{0}$ parallel and perpendicular to the V–N bond. For HYSCORE spectra recorded from the $m_{I}=+3/2_{⊥}$ field setting and that span all orientations of $B_{0}$ in the porphyrin plane, the measured value of A is the average ${\langle A\rangle }_{⊥}\approx \left(A_{//}+A_{⊥}\right)/2$ , with $A_{//}=a_{\mathrm{iso}}+2T$ and $A_{⊥}=a_{\mathrm{iso}}-T$ . Parameters $a_{\mathrm{iso}}$ and T are the isotropic and dipolar hf interactions, respectively. For the corresponding HYSCORE spectrum of Ref 1 recorded with the $m_{I}=-1/2$ field setting (top left spectrum of Fig. 6), where almost all molecular orientations are probed, the hf interaction measured from dq–dq peaks (Eq. 4) is approximated as the average value over all the possible field orientations, given by $\langle A\rangle \approx \left(A_{//}+2A_{⊥}\right)/3$ . The dq–dq peak of VO-P1 at [ -5.3 ; + 9.5] MHz gives $\langle A\rangle =-7.28$ MHz for this field setting. Combining the hf values ${\langle A\rangle }_{⊥}$ and $\langle A\rangle$ measured for the two observing fields shows that the hf interaction is mostly isotropic, with $a_{\mathrm{iso}}=-7.3$ MHz and T<0.1 MHz for VO-P1. By the same procedure, the ${}^{14}$ N parameters of VO-P2 are $a_{\mathrm{iso}}=-6.5$ MHz and T=0.2 MHz. It is interesting to note that the value $a_{\mathrm{iso}}=-7.3$ MHz measured for VO-P1 is close to the value $a_{\mathrm{iso}}=-7.23$ MHz measured by pulse ENDOR for VO-P complexes in heavy crude oil from the Republic of Tatarstan (Russia) and the value $a_{\mathrm{iso}}=-7.2$ MHz measured by pulse EPR for vanadyl octaethylporphyrin (Fukui et al., 1993; Gracheva et al., 2016). This good concordance between measurements of a different nature confirms that a simple measurement of the sharp dq–dq peaks recorded by observing the intense and nearly isotropic $m_{I}=-1/2_{⊥}$ EPR line gives a good estimate of the isotropic hf coupling $\langle A\rangle \approx \left(A_{//}+2A_{⊥}\right)/3=a_{\mathrm{iso}}$ in VO-P complexes.

Vanadyl complex VO-P1 is present in the four studied samples, while the VO-P2 complex is detectable only in Ref 1, Hum 3 and Ref 2 (Figs. 6 and S8). Another complex, referred to as VO-P4 ( $a_{\mathrm{iso}}=-6.8$ MHz and T=0.6 MHz), is also present in An 2 and Hum 3 but is clearly absent in Ref 1 and Ref 2. However, an additional dq–dq peak attributed to a complex VO-P3 ( $a_{\mathrm{iso}}\approx -7.3$ MHz) was detected only in pure bitumen samples Ref 1 and Ref 2 by observing the sharp $m_{I}=-1/2$ EPR transition. As sq–dq transitions could not be detected for VO-P3 and VO-P4, it was not possible to obtain an estimation of $\left|Q\right|$ in these cases. The ${}^{14}$ N parameters of the four VO-P complexes are reported in Table 3. The smaller $a_{\mathrm{iso}}$ values measured for VO-P2 and for VO-P4 do not seem to have equivalents in the literature; however, they are relatively close to those measured by Moons et al. (2017) in vanadyl perfluorophtalocyanine ( -6.9 MHz).

All this interpretation was based on the analysis of only a small portion of each HYSCORE spectrum (rectangular boxes in Fig. 5). This procedure raises the question of the origin of all other correlation peaks and ridges present in the HYSCORE spectra (Fig. 5). To test the validity of the proposed analysis, the whole ${}^{14}$ N HYSCORE spectra of VO-P1 and VO-P2 complexes recorded at the $m_{I}=+3/2_{⊥}$ field setting were simulated with EasySpin software (Stoll and Schweiger, 2006) by adjusting the values of $a_{\mathrm{iso}}$ , T and $Q_{zz}$ (the z component of the quadrupolar interaction). The result is shown in the middle of Fig. 5b and in Fig. S10. Except for peak intensities, which are not correctly accounted for in the simulations, the main features of experimental HYSCORE spectra are well reproduced, taking into account the fact that the shape of the simulated spectra is very sensitive to the values of $a_{\mathrm{iso}}$ , T and $Q_{zz}$ parameters. This validation of nitrogen parameters reported in Table 3 calls for several comments.

The simulated values $Q_{zz}=+0.9$ MHz and +1.0 MHz for VO-P1 and VO-P2 complexes are about twice the experimental values $\left|Q\right|=$ 0.47 and 0.55 MHz measured from sq–dq correlation peaks recorded at the $m_{I}=+3/2_{⊥}$ field setting. As the quadrupolar interaction is a traceless tensor (i.e. $Q_{zz}+Q_{xx}+Q_{yy}=0)$ , this apparent discrepancy could mean that the largest component of the ${}^{14}$ N quadrupolar interaction is nearly perpendicular to the porphyrin plane, so that the experimental values measured here by setting the magnetic field in the porphyrin plane ( $m_{I}=+3/2_{⊥}$ EPR transition) correspond to $\left|Q\right|\approx Q_{xx}\approx Q_{yy}\approx Q_{zz}/2$ .

All the other correlation peaks and ridges visible in the ( + , - ) quadrant are clearly due to the same porphyrinic nitrogen atoms that give rise to the observed dq–dq peaks. Thus it is not necessary to invoke hf interactions with other nuclei ( ${}^{14}$ N, ${}^{13}$ C or others).

Unfortunately, the ( + , +) quadrant does not give any useful information on the ${}^{13}$ C hf interaction because the corresponding peaks are mostly hidden under ${}^{14}$ N correlations which come out in the same frequency range as ${}^{13}$ C ( $\nu_{C}=3.7$ MHz), as shown by the simulations in Figs. 5 and S10. Also, in a multi-spin system such as VO-P complexes, nuclei with weak modulations (such as ${}^{13}$ C and ${}^{1}$ H) can be partially or totally suppressed by nuclei with deep modulations, which is the case with ${}^{14}$ N (Stoll et al., 2005), explaining why it is difficult to measure ${}^{13}$ C hf interactions in VO-P complexes.

### 3.4 Focus on the human mummy Hum 3

This ${}^{1}$ H and ${}^{14}$ N hf analysis of vanadyl probes in black coatings confirms the peculiarity of Hum 3 compared to other samples of black matter, as previously evidenced by cw-EPR (Dutoit et al., 2020). Hum 3 was taken from the neck of the mummy found in Nehemsimontou's coffin (25th Dynasty, 744 to 656 BC), purchased in 1837 by the museum of Boulogne (France) from a private collector. It later turned out that the mummy and the coffin had been assembled for the purpose of a better sale, a common practice in the 19th century. This beautiful mummy is covered with a solid, black and shiny substance and has therefore an unknown origin (Fig. 1). Cw-EPR and GC-MS analysis showed that this black coating is made of pure bitumen (Dutoit et al., 2020). Contrary to other black coatings studied in this work (animal and human mummies, coffin), Hum 3 contains no VO-nP (non-porphyrinic vanadyl complexes), and its EPR spectrum is very similar to that of pure bitumen (Ref 1 and Ref 2) (Dutoit et al., 2020). This similarity to native bitumen is confirmed by the almost identical ENDOR spectra of Hum 3, Ref 1 and Ref 2 (Figs. 4 and S5). These samples do not exhibit the intense ${}^{1}$ H matrix line present when natural substances are mixed with bitumen, which is consistent with the fact that Hum 3 was made of pure bitumen.

The similarities and differences between the Dead Sea asphalt (Ref 1) and the black coatings of mummies are documented with more precision by the analysis of the nuclear transitions of ${}^{14}$ N. Among the three vanadyl porphyrin complexes VO-P1, VO-P2 and VO-P3 detected in Ref 1 (but also in the commercial Judea bitumen Ref 2), VO-P1 and VO-P2 are also present in *Hum3*, while only VO-P1 was detected in the animal mummy An 2. As the latter contains less than 20 % of the VO-P content of Ref 1, it is possible that VO-P2 peak intensity may be too low to be detected in this case. Bitumen from mummies An 2 and Hum 3 also contains a fourth complex VO-P4 that is absent in the reference bitumen samples Ref 1 and Ref 2. Instead, Ref 1 and Ref 2 are characterized by the presence of the VO-P3 complex. As the preparation of the embalming coating by ancient Egyptian implies that bitumen was heated to the liquid state in order to be mixed with the other ingredients and to spread on the mummy, we may hypothesize that VO-P4 originates from the thermal transformation of an unstable complex VO-P3 initially present in natural bitumen. Laboratory experiments will be necessary to test this hypothesis.

## 4 Conclusion and perspective

In summary, this work shows that vanadyl porphyrin (VO-P) complexes commonly found at trace level in natural bitumen and oil can be used as intrinsic paramagnetic probes for a non-destructive analysis of the black coatings covering ancient Egyptian mummies and funerary artifacts. In a previous study by cw-EPR (Dutoit et al., 2020), we had shown that even small quantities of bitumen (relative to other organic substances) in these black coatings could be easily detected by the joint presence of VO-P and carbon radicals (C ${}^{0})$ characteristic of fossil organic matter. Unlike conventional micro-destructive molecular analysis techniques (GC-MS), which often minimize the presence of bitumen in black coatings (Lucejko et al., 2017), EPR is non-destructive as samples are analysed directly without preliminary physical or chemical treatment, and even a small amount of bitumen in a coating can be unambiguously detected.

In the present work, additional information on the nanostructure of the black coatings and on the speciation of vanadyl porphyrins was obtained by hyperfine spectroscopy (ENDOR, HYSCORE). The ${}^{1}$ H-ENDOR spectra reveal that the amplitude of the matrix line (representing distant hydrogen atoms) regularly increases with decreasing amount of bitumen in the black coating. This regular variation has been modeled and indicates that all black coatings have similar nanostructures, with nano-sized aggregates of bitumen embedded in a matrix of bioorganic substances (conifer resin, fat, wax). These similarities in nanostructures may reflect a similarity in preparation recipes of black coatings in various funerary contexts (animal and human mummies, coffins) dating from the Late Period to the Greco–Roman period of the history of ancient Egypt. However, this hypothesis needs to be tested by studying a larger body of archaeological samples and by performing laboratory reconstructions of black coatings. This should ultimately make it possible to specify the manufacturing recipes used in ancient Egypt. The speciation of VO-P was studied by detecting ${}^{14}$ N nuclear transitions of porphyrins by HYSCORE spectroscopy. At least four types of VO-P complexes were identified from analysis of the double-quantum (dq–dq) correlation peaks of ${}^{14}$ N. These sharp peaks are relatively easy to detect even if the bitumen is mixed with other natural substances. Two of these VO-P complexes (VO-P1 and VO-P2) are present in both the reference bitumen samples and the coatings of the two human and animal mummies that we were able to study. The structure of a third type of VO-P present in reference bitumen samples (VO-P3) appears to have been transformed into another type (VO-P4) during the preparation of the coating of the two mummies. This indicates that some VO-P complexes may be thermally or chemically unstable, and their identification could give information on the thermal/chemical treatments employed in ancient Egypt for the preparation of black coatings. This work and Dutoit et al. (2020) show that combining various EPR techniques (cw-EPR, ENDOR, HYSCORE) is a promising tool for a non-destructive exploration of the nanostructure and composition of black coatings of ancient Egyptian mummies and funerary artifacts. This methodology should also allow us to better apprehend local economies, workshop practices and recipes, supply areas as well as trade routes of bituminous materials in the past.

## Supplement

10.5194/mr-3-111-2022-supplement Samples; EPR spectra; ENDOR spectra; derivation of Eq. (1); HYSCORE spectra; estimation of second-order contributions to the 14N parameters from dq–dq and sq–dq correlation peaks. The supplement related to this article is available online at: https://doi.org/10.5194/mr-3-111-2022-supplement.

## Data Availability

EPR raw and processed data have been deposited in Mendeley data repository available at https://doi.org/10.17632/bnb9jsjs5r.1 (Dutoit et al., 2022).

## Code availability

The Easyspin package for EPR simulation is available at https://www.easyspin.org/download.html (Stoll and Schweiger, 2006). Matlab is a commercial software from MathWorks.
