# Supplementary material for: Insight into the structure of black coatings of ancient Egyptian mummies by advanced electron magnetic resonance of vanadyl complexes
Source: Magn Reson (Gott). 2022 Jul 13;3(2):111–24. doi: 10.5194/mr-3-111-2022 (PMC10539842; doi:10.5194/mr-3-111-2022)
Supplement: Samples; EPR spectra; ENDOR spectra; derivation of Eq. (1); HYSCORE spectra; estimation of second-order contributions to the 14N parameters from dq–dq and sq–dq correlation peaks. The supplement related to this article is available online at: https://doi.org/10.5194/mr-3-111-2022-supplement. [file mr-3-111-supplement.pdf]

Supplement of Magn. Reson., 3, 111–124, 2022
https://doi.org/10.5194/mr-3-111-2022-supplement
© Author(s) 2022. CC BY 4.0 License.

Supplement of

Insight into the structure of black coatings of ancient Egyptian mummies by advanced electron magnetic resonance of vanadyl complexes

Charles E. Dutoit et al.

Correspondence to: Didier Gourier (didier.gourier@chimieparistech.psl.eu)

The copyright of individual parts of the supplement might differ from the article licence.

S1 Samples

Table S1. Description of samples

Samples	Object	Provenance	Origin /Dating	Description
Ref 1	Natural asphalt	C2RMF	Dead Sea, floating blocks (Late Cretaceous)	Black solid
Ref 2	Bitumen of Judea	C2RMF	Commercial	Brown powder
Hum 1	Anthropomorphic coffin	The Art and History museum of Narbonne, France (Ref: C2RMF76267)	Upper Egypt (Abydos ?). Ptolemaïc period (332 BC – 30 BC)	Coffin of Irethorerou, servant of Khonsou, of the White Crown and of Horus. Black matter covering the bottom of the coffin
Hum 2	Human mummy	The Hieron museum, Paray-le-Monial, France (Ref: FZ30827)	Late Period, end of the IVth century BC	Mummy of a 35-45 years old man, named …djeb. Set of crossed bands, coated with dark matter
Hum 3	Human mummy	Museum of Boulogne, France (Ref. 35906)	Late period, XXVth dynasty (744 BC - 656 BC)	Mummy found in the coffin of Nehemsimontou, coated with black matter.
An 1	Ram mummy	The Louvre museum, Paris, France; (Ref: C2RMF 64621)	Upper Egypt (Elephantine). Late period (672 BC–322 BC)	Fragment of black matter covering the mummy
An 2	Ram mummy	The Thomas Dobrée museum, Nantes, France (Ref: C2RMF36230)	Upper Egypt. Late Period (664 BC – 332 BC).	Fragments of black matter covering the mummy.
An 3	Ram mummy (the same as **An 2**)	The Thomas Dobrée museum, Nantes, France (Ref: C2RMF36230)	Upper Egypt. Late Period (664 BC – 332 BC).	fragments of tissue strips covering the mummy, coated with a brown material
An 4	Crocodile mummy	Musée des confluences, Lyon, France (Ref : 90001841)	Upper Egypt (Kom Ombo). Ptolemaïc period	Posterior part of mummified crocodile skull, covered with black matter.

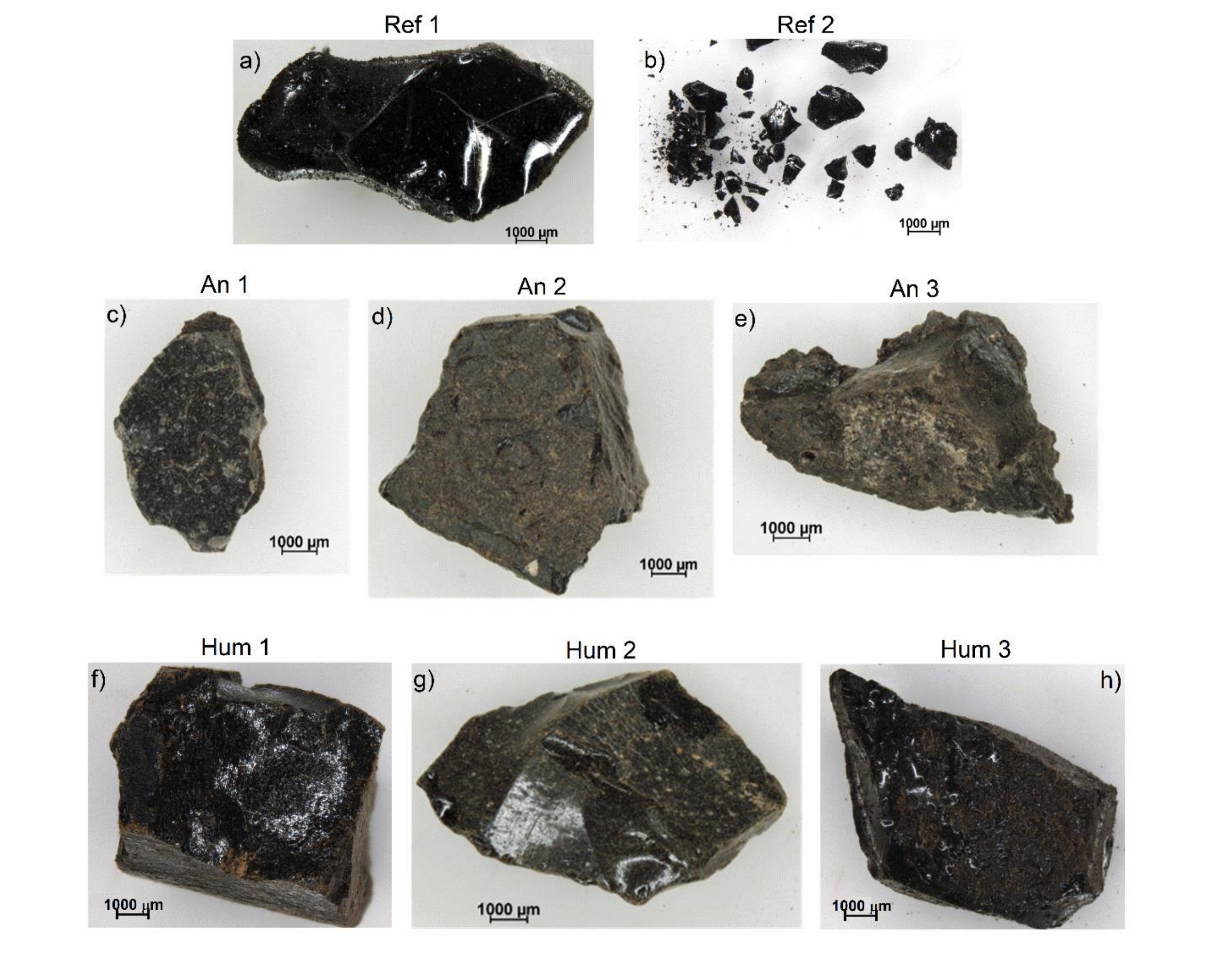

Figure S1. Binocular photographs of the samples studied in this work. © C2RMF.

S2 EPR spectra:

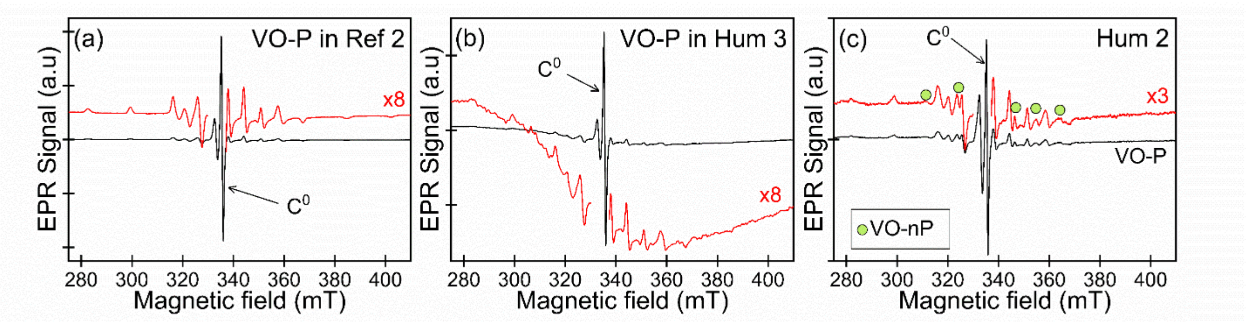

Figure S2. EPR spectra at X band and at room temperature of bitumen reference and human mummies: (a) ***Ref 2***, (b) ***Hum 3*** and (c) ***Hum 2***. This highlights the lack of VO-nP complexes (green circles) in ***Ref 2*** and ***Hum 3***.(From Dutoit, et al., 2020)

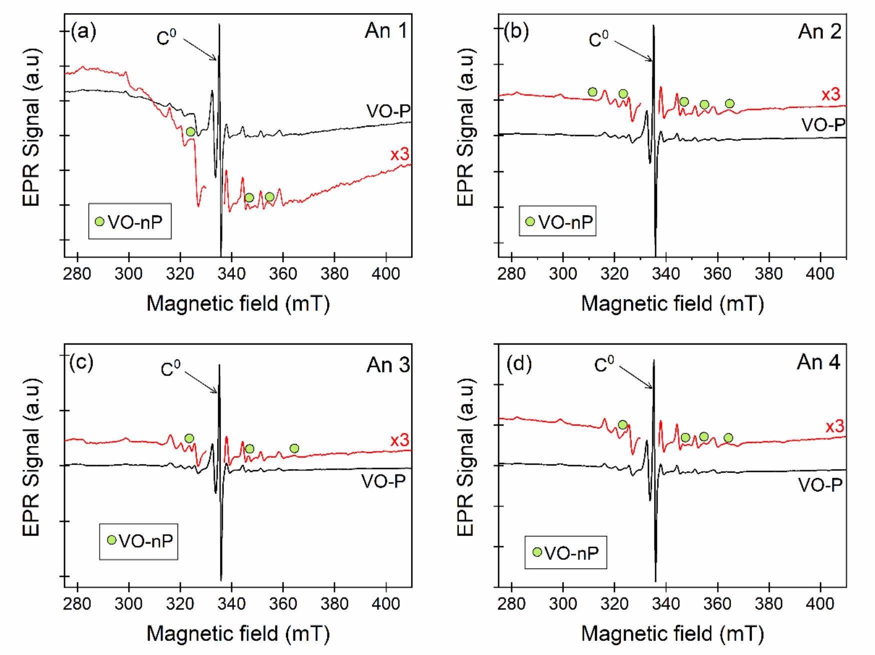

Figure S3. EPR spectra at X band and at room temperature of animal mummies, highlighting EPR lines of VO-nP complexes in (green circles). (From Dutoit, et al., 2020)

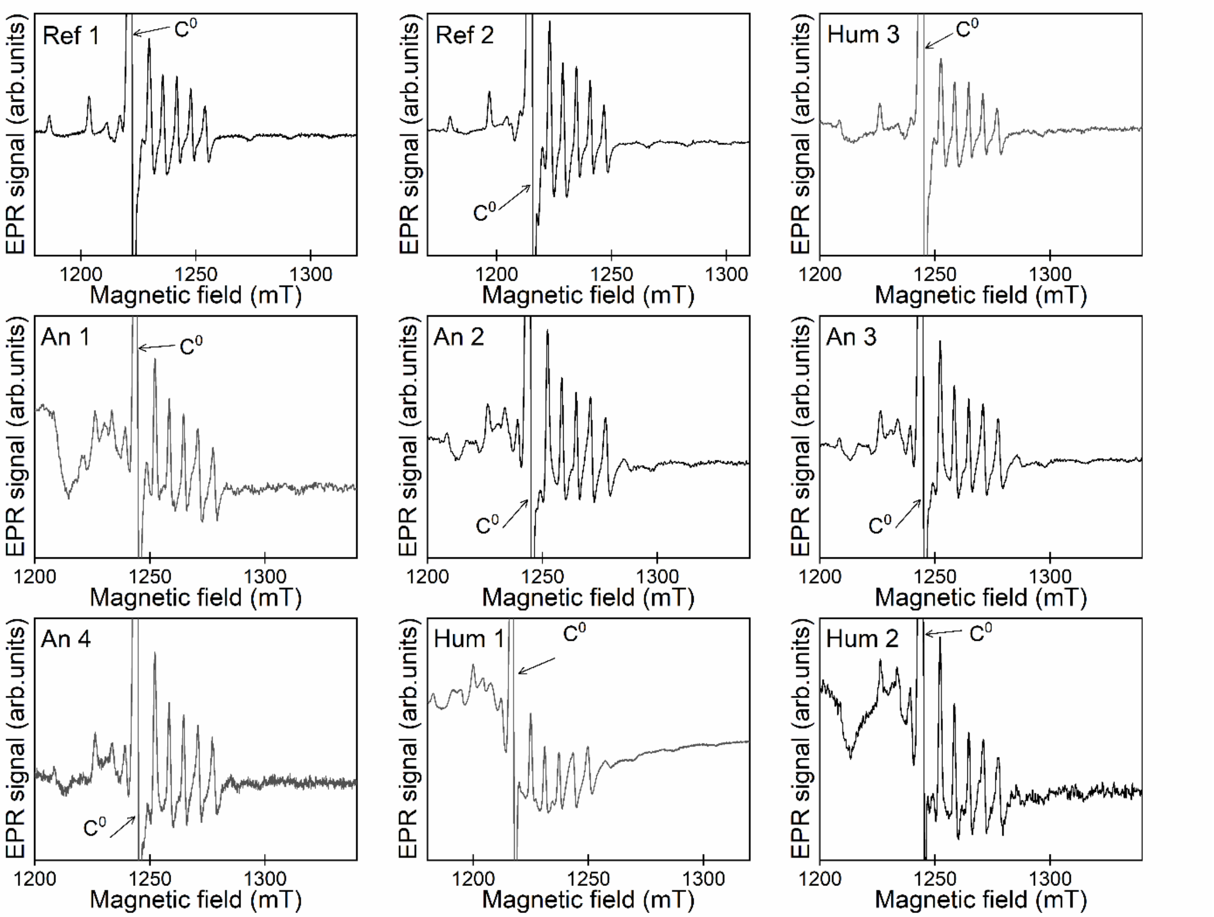

Figure S4. EPR spectra at Q band and at 100 K of the reference bitumen and samples of black coatings.

S3 ENDOR spectra

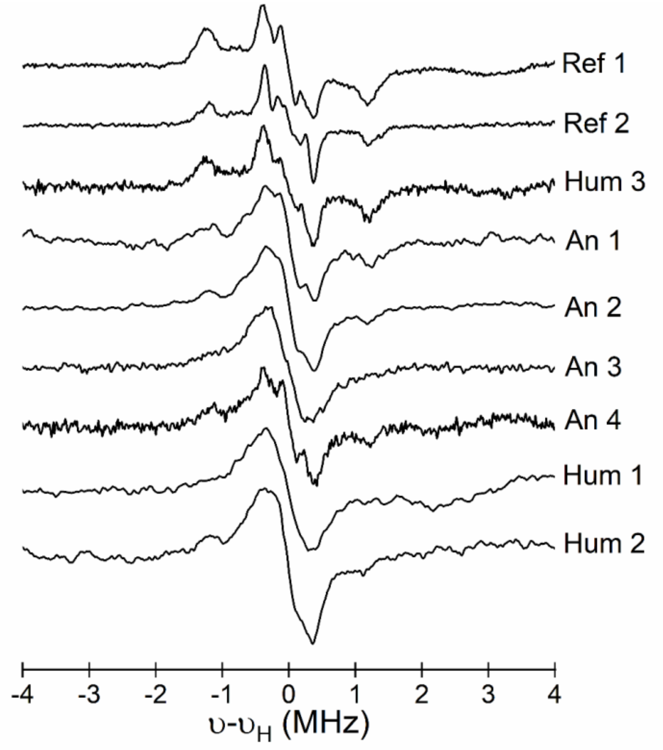

Figure S5. ^{1}H ENDOR spectra at Q band and at 100K of the reference bitumen and samples of black coatings. The field setting values are 1230.9 (***Ref 1***), 1225.4 mT (***Ref 2***), 1230.6 mT (***Hum 3***), 1253.2 mT (***An 1***), 1252.8 mT (***An 2***), 1228.1 (***An 3***), 1253.1 mT (***An 4***), 1222.7 mT (***Hum 1***) and 1252.9 mT (***Hum 2***).

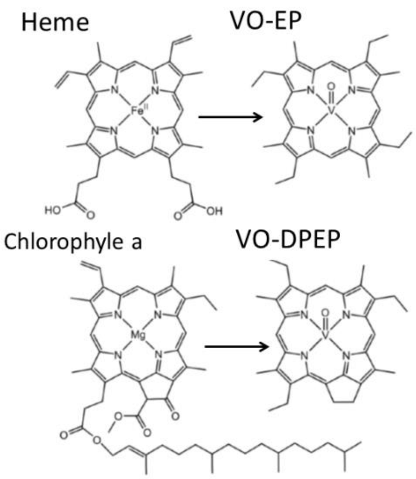

Figure S6. Two examples of geoporphyrins commonly found in oil, with the corresponding parent biomolecules

S4 Derivation of Equation 1

The ^{1}H ENDOR spectrum is the superposition of two independent signals: (i) one from the protons of the C-H$_{meso}$ bridges linking pyrole groups of porphyrin ligands, hereafeter referred to as VOP-^{1}H, and (ii) the other one from the matrix protons, hereafeter referred to as M-^{1}H, corresponding to protons of asphaltene, of the natural substances of the black matter, and of protons of alkyl substituent in porphyrin ligands. M-^{1}H protons are characterized by a pure dipolar hf interaction while VOP-^{1}H protons are characterized by an isotropic hf interaction in addition to the dipolar one.

Let X be the signal height at the frequency $\nu_{\parallel}$ corresponding to the parallel component of the VOP-^{1}H signal and Y the signal height at the maximum of perpendicular component of the VOP-^{1}H at frequency $\nu_{\perp}$ (see Fig.4a). Let also X_{VOP}, X_M, Y_{VOP} and Y_M be the respective contributions of a *single* VOP molecule and a *single* M-^{1}H to X and Y. Then :

$$\frac{X}{Y} = \frac{N_{VOP} X_{VOP} + N_M X_M}{N_{VOP} Y_{VOP} + N_M Y_M} \qquad \text{(S1)}$$

where N_{VOP} and N_M are the total numbers of VOP molecules and matrix protons in the sample, respectively.

The VOP molecules are embedded in bitumen aggregates spread within a bioorganic matrix, which contains the M-^{1}H's. As the M-^{1}H's are detected upon saturating an EPR transition of the VOP molecules, they must have a residual dipolar hf interaction with the VOP's. We thus assume that the detected M-^{1}H's are in a layer of volume V_L surrounding a bitumen aggregate (Fig. S7), then $N_M = N_A V_L [H]$ where N_A is the total number of bitumen aggregate in the sample and $[H]$ the concentration of M-^{1}H's in the matrix. We also have $N_{VOP} = [VOP]V$, with $[VOP]$ the concentration of VOP's in the sample and V, the sample volume. As the experimental variable is $x = [VOP]/[VOP]_{ref}$, where $[VOP]_{ref}$ is the VOP concentration in the reference sample ***Ref 1***, N_{VOP} is then rewritten as $N_{VOP} = x[VOP]_{ref}V$, yielding: $\frac{N_M}{N_{VOP}} = \frac{a}{x}$, with $a = N_A \frac{V_L}{V} \frac{[H]}{[VOP]_{ref}}$. Finally, we obtain:

$$\frac{X}{Y} = \frac{X_{VOP}}{Y_{VOP}} \times \frac{x + aX_M / X_{VOP}}{x + aY_M / Y_{VOP}} \qquad \text{(S2)}$$

From the ENDOR spectrum of ***Ref 1*** dominantly made of the contribution of VOP-^{1}H signal and negligible contribution from M-^{1}H, we get $\frac{X_{VOP}}{Y_{VOP}} \approx 0.625$ and assuming a gaussian lineshape for the M-^{1}H ENDOR line, we get $\frac{X_M}{Y_M} \approx 0.03$ giving $\frac{X_M}{X_{VOP}} \approx 0.048 \frac{Y_M}{Y_{VOP}}$ and finally:

$$\frac{X}{Y} \approx 0.625 \times \frac{x + 0.048 \times b}{x + b} \tag{S3}$$

with a single adjustable parameter $b = a\frac{Y_M}{Y_{VOP}}$, which depends on the sizes and dispersion of the bitumen aggregates through N_A and the ratio $\frac{V_L}{V}$.

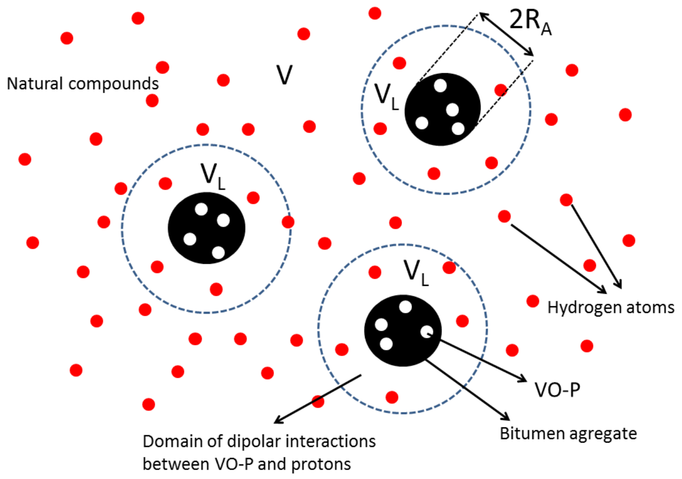

Figure S7. Schematic description of a bitumen aggregate in interaction with protons of bioorganic compounds.

S5 HYSCORE spectra of *Ref 2*

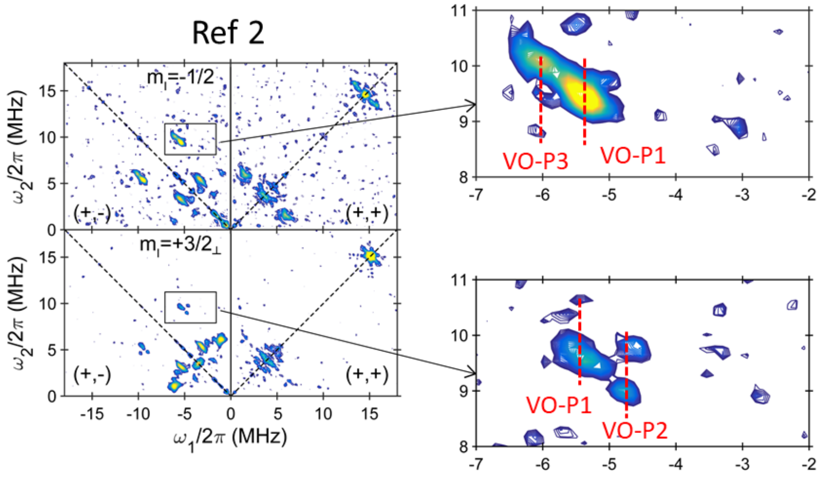

Figure S8. HYSCORE spectra of ***Ref 2*** recorded by observing the two EPR transitions m_I = -1/2 and m_I = +3/2⊥. Figures on the right show the portions of spectra corresponding to the frequency range of dq-dq correlations. Correlations sq-dq are not clearly detected because the VO-P content is lower in ***Ref 2*** than in ***Ref 1***.

S6 Simulation of HYSCORE spectra

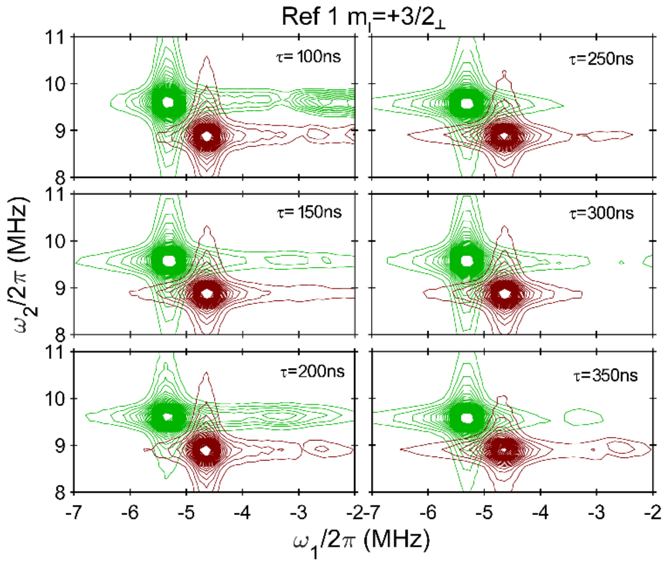

Figure S9. Effect of τ values on simulated dq-dq correlation peaks for VO-P1 (in green) and VO-P2 (in red) complexes, showing the lack of blind spot effects. Field setting m_I = +3/2⊥ at 355.6 mT

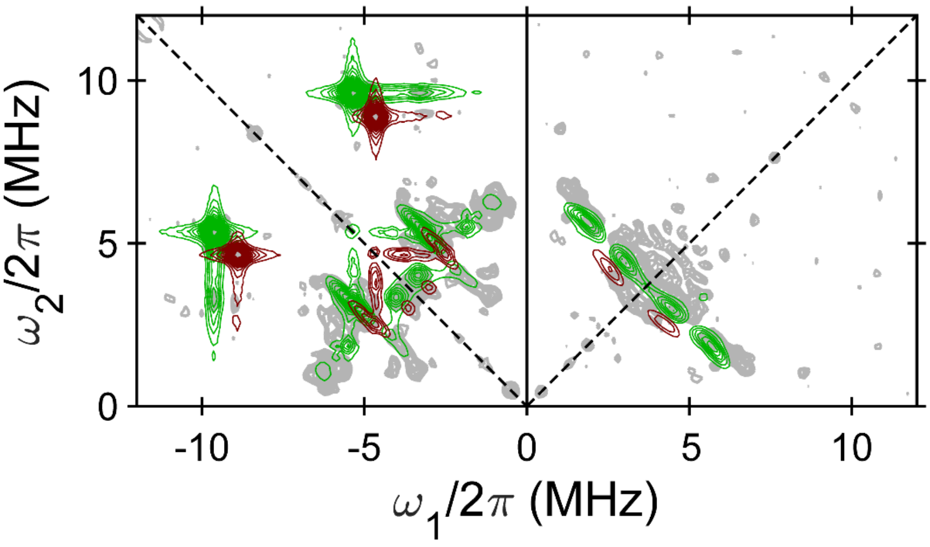

Figure S10. Comparison of experimental and simulated HYSCORE spectra of VO-P1 and VO-P2 of sample Ref 1. The experimental spectrum is in grey, the simulated spectra are in green (VO-P1) and red (VO-P2). Field setting m_I = +3/2⊥ at 355.6 mT. The simulation parameters are given in Table 3

S7 Estimation of second order contributions in ^{14}N parameters from dq-dq and sq-dq correlation peaks

The first order nuclear spin energy levels of a single m_s state of VO^{2+} interacting with the nuclear spin I = 1 of a ^{14}N nucleus is given by:

$$E = \pm\frac{1}{2}Am_I + Q\left(\frac{3}{2}m_I^2 - 1\right) - \nu_N m_I \tag{S4}$$

where the energy E, the hf interaction A and the quadrupolar interaction Q are taken along the direction of the magnetic field. The corresponding energy level diagram is given in Fig. S9 for the two m_s states. The frequencies of the single quantum ($\Delta m_I = \pm 1$) and double quantum ($\Delta m_I = \pm 2$) nuclear spin transitions of ^{14}N are given by (Reijerse, et al., 1998; Dikanov, et al., 2004):

$$\begin{aligned} \nu_{1sq}^{\pm} &= \frac{A}{2} \pm \nu_N + \frac{3Q}{2} + (2^{\text{nd}}\ \text{order terms}) \\ \nu_{2sq}^{\pm} &= \frac{A}{2} \pm \nu_N - \frac{3Q}{2} + (2^{\text{nd}}\ \text{order terms}) \end{aligned} \tag{S5}$$

$$\nu_{dq}^{\pm} = A \pm 2\nu_N + \frac{\mathrm{A}^{(2)}}{(A/2) \pm \nu_N} \tag{S6}$$

The second order corrections $\nu^{(2)}$ to the single quantum frequencies $\nu_{1sq}^{\pm}$ and $\nu_{2sq}^{\pm}$ are:

$$\nu_{1,2\mathrm{sq}}^{(2)+} = \left(A^{(2)} \pm Q^{(2)}\right) / \left(A - 2\nu_N\right)$$
$$\nu_{1,2\mathrm{sq}}^{(2)-} = \left(A^{(2)} \pm Q^{(2)}\right) / \left(A + 2\nu_N\right) \quad \text{(S7)}$$

where

$$A^{(2)} = 1/4\left(T_{np}^2 + T_{nq}^2\right) + K^2\left(3 + \eta^2\right) - 3Q_n^2 / 4$$
$$Q^{(2)} = 3\left(Q_{np}T_{np} + Q_{nq}T_{nq}\right) \quad \text{(S8)}$$

$K = e^2qQ/4h$ is the quadrupolar coupling constant. The matrix elements T_{np} and T_{nq} in $A^{(2)}$ and $Q^{(2)}$ are anisotropic components of the hf interaction, **n** is the orientation of the magnetic field, and **p** and **q** are two orientations perpendicular to **n** and to each other.

Determination of *A* from expressions of $\nu_{dq}^{\pm}$ is not affected by 2nd order correction:

$$A = \frac{2\nu_N\left(\nu_{dq}^{+} + \nu_{dq}^{-}\right)}{8\nu_N - \left(\nu_{dq}^{+} - \nu_{dq}^{-}\right)} \quad \text{(S9)}$$

On the contrary, measurement of *Q* from expressions from Eqs.S5 is affected by second order corrections and necessitates the preliminary determination of $\nu_{1sq}^{\pm}$ and $\nu_{2sq}^{\pm}$. As only a part of the sq-dq correlations has been detected, only half of then sq frequencies could be determined precisely. The observed sq-dq correlations for VO-P1 and VO-P2 correlate the dq transition of the m_s = +1/2 state with one of the sq transitions of the m_s = -1/2 state, and recalling that $\nu_{1sq}^{-} + \nu_{2sq}^{-} = \nu_{dq}^{-}$, all transitions in the m_s = -1/2 state are known without uncertainty due to 2nd order corrections. Single-quantum transitions in the m_s =+1/2 state were obtained to 1st order by the equation $\nu_{1sq}^{+} - \nu_{1sq}^{-} \approx \nu_{2sq}^{+} - \nu_{2sq}^{-} \approx 2\nu_N$ and are thus affected by 2nd order corrections. The resulting diagrams for VO-P complexes are given in Fig.S9. In the absence of unambiguous sq-dq correlations for VO-P3 and VO-P4, we could not obtain sq frequencies and quadrupolar parameter *Q* for these complexes.

The second order term in Eqs. S5, S6, S7 and S8 can be estimated as follows. Combining the two dq frequencies gives:

$$\nu_{dq}^{+} - \nu_{dq}^{-} = 4\nu_N - \frac{2\nu_\mathrm{n}\mathrm{A}^{(2)}}{A^2/4 - \nu_N^2} \quad \text{(S10)}$$

From the experimental values of $\nu_{dq}^{\pm}$ and from ν_N = 1.1 MHz, we obtain $\mathrm{A}^{(2)}$ = 0.55 MHz and 0.44 MHz in VO-P1 and VO-P2, respectively. This gives a second order contribution $\frac{\mathrm{A}^{(2)}}{(A/2) \pm \nu_N} \approx$ 0.1 – 0.2 MHz in Eq. S6 for VO-P complexes, which corresponds also to the uncertainty in the experimental measurement of dq frequencies in Fig. 6.

Concerning second order contributions in the determination of the quadrupolar interaction *Q*, expressions for sq frequencies give $\nu_{1sq}^{+} - \nu_{2sq}^{+} = 3Q + \frac{Q^{(2)}}{A/2 + \nu_N}$ and $\nu_{1sq}^{-} - \nu_{2sq}^{-} = 3Q + \frac{Q^{(2)}}{A/2 - \nu_N}$,

which gives an estimation $Q^{(2)} \approx 0.5$ MHz of the same order as $\mathrm{A}^{(2)}$, and thus $\frac{Q^{(2)}}{A/2 \pm \nu_N} \approx 0.1 - 0.2$ MHz.

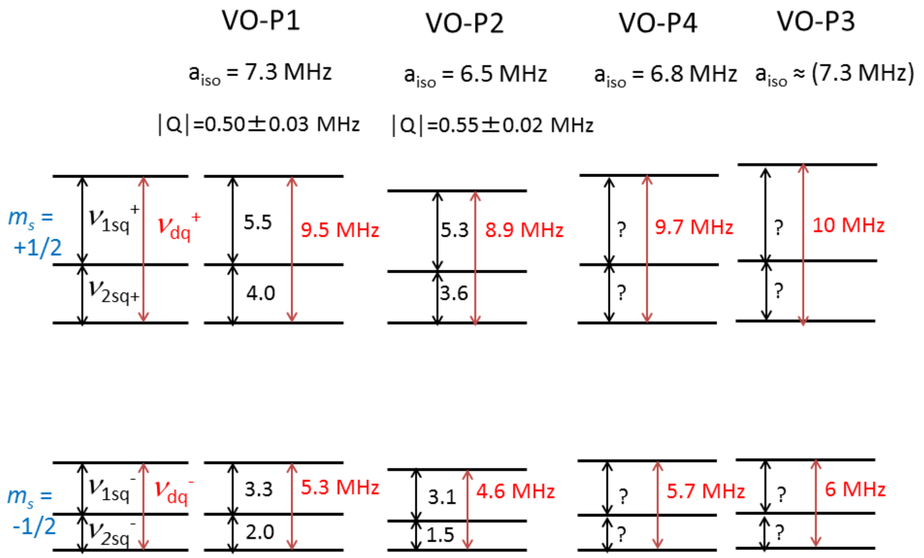

Figure S11. Energy level diagram of an electron spin S = 1/2 interacting with a nuclear spin I = 1, showing single quantum (sq) transitions (in black) and double quantum (dq) transitions (in red), with the corresponding diagrams for the four VO-Ps detected in the black matter; the four experimental diagrams correspond to the observation of the EPR transition m_I = +3/2⊥ (for VO-P1, VO-P2 and VO-P4) and m_I = -1/2 for VO-P3; the quadrupolar interaction can be measured from sq transitions only when sq-dq peaks are detectable (VO-P1 and VO-P2); a_{iso} was deduced from dq-dq transitions obtained with the two EPR transitions m_I = -1/2 and m_I = +3/2⊥.
